# Genetic Affinities between Trans-Oceanic Populations of Non-Buoyant Macroalgae in the High Latitudes of the Southern Hemisphere

**DOI:** 10.1371/journal.pone.0069138

**Published:** 2013-07-22

**Authors:** Ceridwen I. Fraser, Giuseppe C. Zuccarello, Hamish G. Spencer, Laura C. Salvatore, Gabriella R. Garcia, Jonathan M. Waters

**Affiliations:** 1 Allan Wilson Centre for Molecular Ecology and Evolution, Department of Zoology, University of Otago, Dunedin, New Zealand; 2 School of Biological Sciences, Victoria University of Wellington, Wellington, New Zealand; 3 Department of Zoology, University of Otago, Dunedin, New Zealand; University of Otago, New Zealand

## Abstract

Marine biologists and biogeographers have long been puzzled by apparently non-dispersive coastal taxa that nonetheless have extensive transoceanic distributions. We here carried out a broad-scale phylogeographic study to test whether two widespread Southern Hemisphere species of non-buoyant littoral macroalgae are capable of long-distance dispersal. Samples were collected from along the coasts of southern Chile, New Zealand and several subAntarctic islands, with the focus on high latitude populations in the path of the Antarctic Circumpolar Current or West Wind Drift. We targeted two widespread littoral macroalgal species: the brown alga *Adenocystisutricularis* (Ectocarpales, Heterokontophyta) and the red alga *Bostrychia* *intricata* (Ceramiales, Rhodophyta). Phylogenetic analyses were performed using partial mitochondrial (COI), chloroplast (*rbc*L) and ribosomal nuclear (LSU / 28S) DNA sequence data. Numerous deeply-divergent clades were resolved across all markers in each of the target species, but close phylogenetic relationships – even shared haplotypes – were observed among some populations separated by large oceanic distances. Despite not being particularly buoyant, both *Adenocystisutricularis* and *Bostrychia* *intricata* thus show genetic signatures of recent dispersal across vast oceanic distances, presumably by attachment to floating substrata such as wood or buoyant macroalgae.

## Introduction

The role of oceanic rafting in structuring littoral and near-shore ecosystems is increasingly being demonstrated through molecular analyses, e.g., [1–7], and by ecological or observational studies, e.g., [8–10]; see also reviews [11–13]:. In particular, detached buoyant macroalgae can form rafts, facilitating trans-oceanic dispersal of themselves and algal-associated fauna [5]. The dispersal ability of buoyant macroalgal species such as southern bull-kelp ( *Durvillaeaantarctica* ) and giant bladder kelp ( *Macrocystispyrifera* ) is highlighted by their broad distributions [14,15]. If strong buoyancy enables some macroalgal taxa to traverse vast oceanic distances, non-buoyant taxa with poorly-dispersive gametes and spores might be expected to show both relatively restricted ranges and poor genetic connectivity; yet many such species have surprisingly broad distributions [16,17] and may therefore be able to disperse long distances [18,19].

There is thus a paradox between the broad distributions of some species and their inherently poor dispersal capacity [20]. Although marine biogeographic studies are increasingly revealing examples of this paradox, we still do not fully understand the factors that favour long-distance dispersal in some species over others, nor how common dispersal capacity is within and among closely related groups [17]. Using multi-gene (mtDNA: COI, cpDNA: *rbc*L, and rRNA: LSU) phylogeographic analyses of two widespread, nonbuoyant subantarctic macroalgal taxa, we here tested the hypothesis that coastal species with low inherent dispersal capacity can nonetheless readily achieve long-distance, trans-oceanic dispersal. We further aimed to assess whether either of the two species, or lineages within them, appear more or less capable of such dispersal. The first of these taxa, *Bostrychia* *intricata* (Bory de Saint-Vincent) Montagne, is a turfing red alga (Rhodophyta) which reproduces sexually, grows in the upper limits of the intertidal zone on rock platforms, and forms dense moss-like mats of intertwined individuals that are particularly common in and around shaded rock cracks and crevices ( [21]: as *Stictosiphonia* *hookeri* ) on numerous subantarctic islands, as well as the coasts of southern New Zealand, Chile and Argentina and the Antarctica Peninsula. *Bostrychia* tetraspores, although slightly motile [22], are short-lived, surviving only about one day in culture (J. A. West, *pers. comm.*), and are thus unlikely to disperse long distances. The second taxon, *Adenocystisutricularis* (Bory de Saint-Vincent) Skottsberg, is a brown macroalga (Phaeophyceae), with a similar geographical range. It also has short-lived gametes, with female gametes settling on the substratum within 30 minutes of release, and the more motile male gametes settling within 24 hours [23]. This species has a biphasic life history, existing as diploid macroscopic sporophytes (tear-drop shaped sacs a few centimetres long) in summer, or solely as thin haploid filaments in winter, and can reproduce either sexually or asexually [23]. Thus, neither species has a life history that seems likely to promote unassisted trans-oceanic dispersal of propagules.

This study does not attempt to provide a comprehensive biogeography of the two target species throughout their entire distributional ranges, but rather focuses on samples collected from high-latitude Southern Hemisphere locations including southern Chile, New Zealand and several sub-Antarctic islands. These disjunct regions are connected oceanographically by the Antarctic Circumpolar Current, and as some genetic affinities of populations of buoyant macroalgae [14,15] and associated invertebrates [4] have previously been shown across vast oceanic distances in these regions, they constitute an ideal system in which to test our hypothesis that non-buoyant algal species can also achieve long-distance dispersal in this setting.

## Materials and Methods

### Sample collection, DNA extraction and sequencing

**Figure 1:**
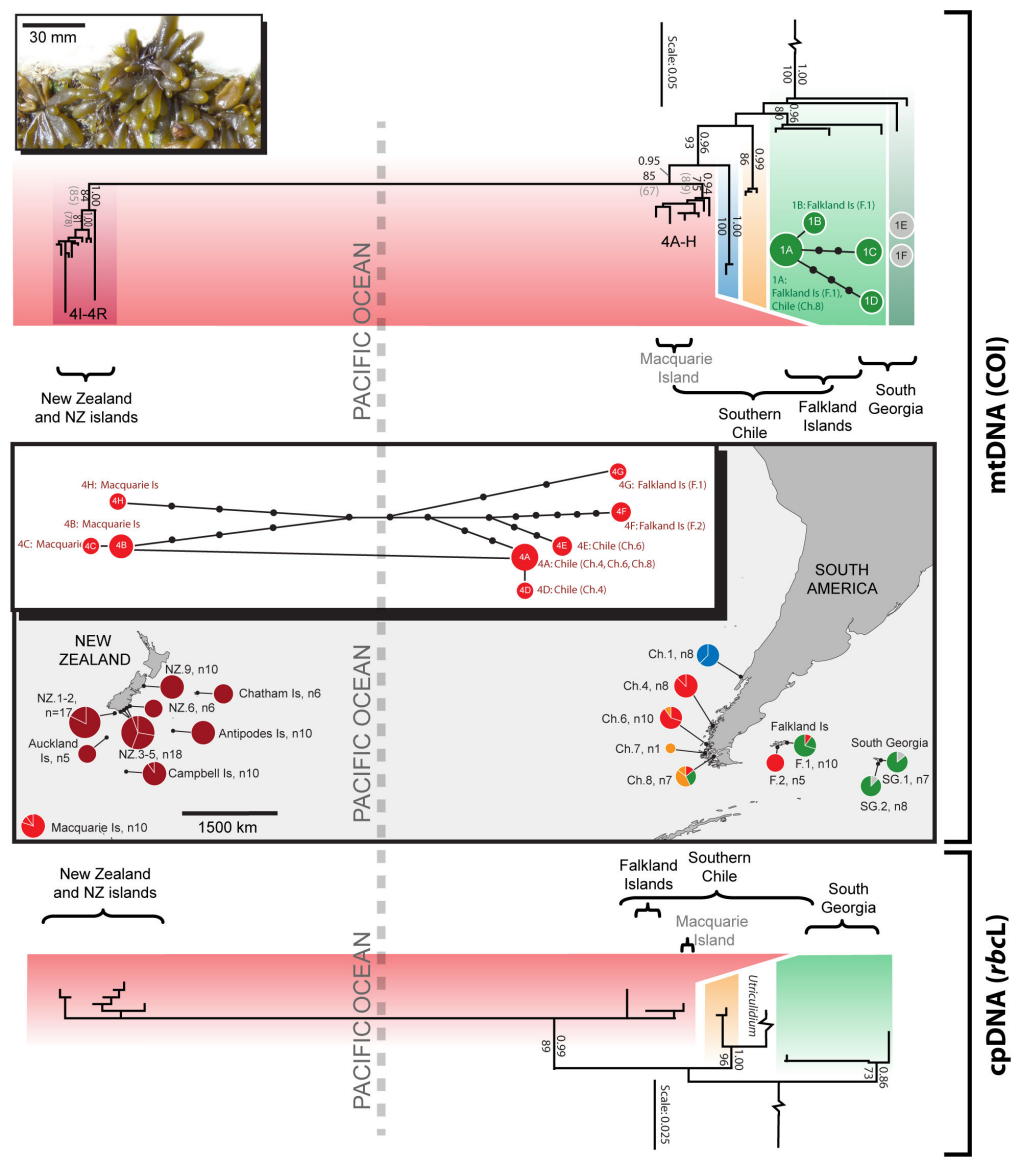
Phylogeographic relationships within *Adenocystisutricularis* based on COI and *rbc*L data. The phylogenetic trees indicate relationships among, and diversity within, major clades or lineage groups (distinguished by colour); support for these clades is shown by Bayesian Posterior Probability values above branches, and ML bootstraps below (those in parentheses show ML bootstraps of >50% for the concatenated analysis of all markers). Outgroup taxa have been removed for clarity. The map indicates clade distributions and proportions at each locality, with multiple haplotypes at a site indicated by pie divisions.

**Figure 2:**
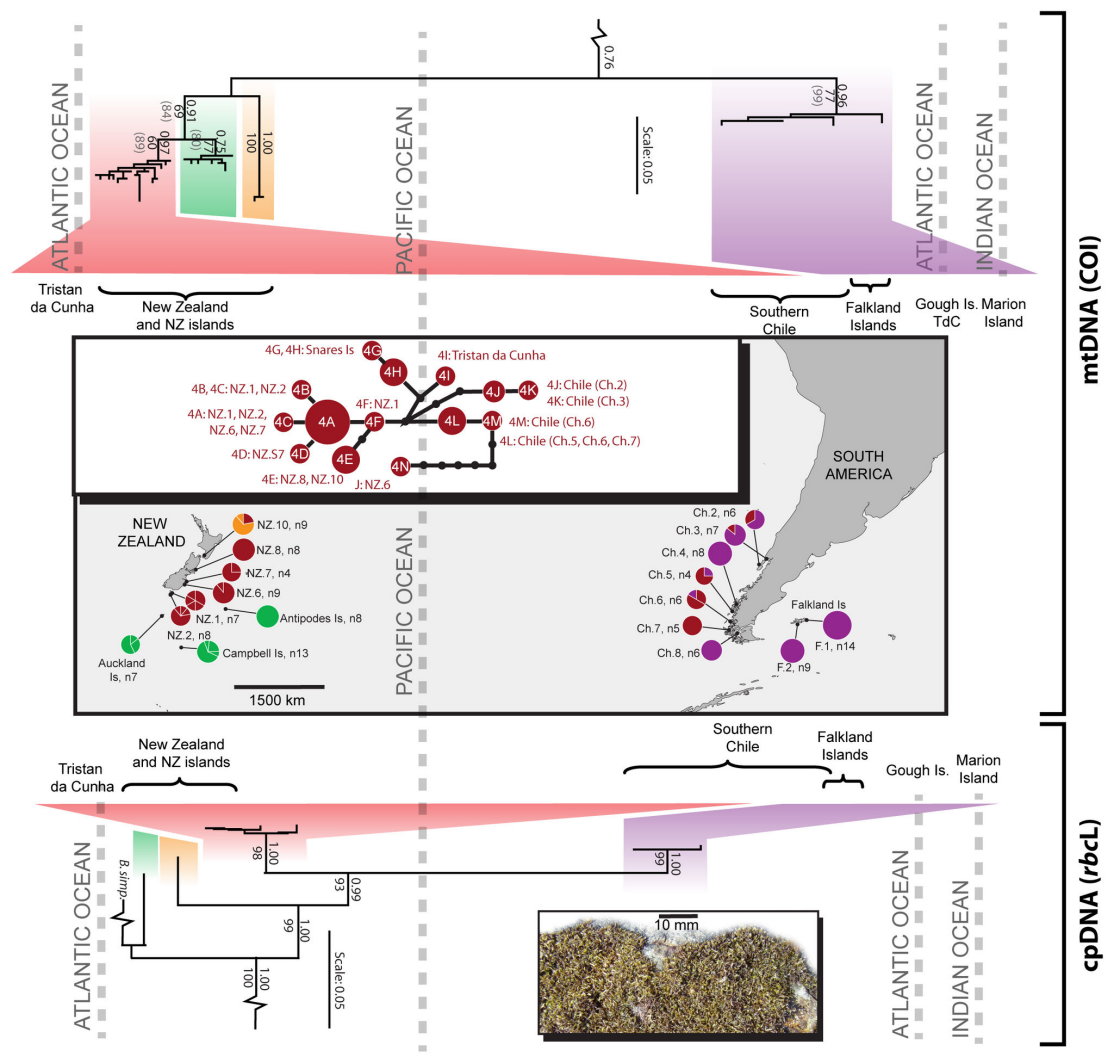
Phylogeographic relationships within *Bostrychia* *intricata* based on COI and *rbc*L data. The phylogenetic trees indicate relationships among, and diversity within, major clades or lineage groups (distinguished by colour); support for these clades is shown by Bayesian Posterior Probability values above branches, and ML bootstraps below (those in parentheses show ML bootstraps of >50% for the concatenated analysis of all markers). Outgroup taxa have been removed for clarity. The map indicates clade distributions and proportions at each locality, with multiple haplotypes at a site indicated by pie divisions.

Samples were collected by hand from intertidal rock platforms around New Zealand, several subantarctic islands, southern Chile, and the Falkland Islands. Sampling sites are indicated on the maps in Figures 1 and 2; site co-ordinates and the number of samples sequenced for each marker from each site are provided in Table S1. Samples were identified in the field based on morphological features, and each was separated from the others by at least 30 cm to ensure that separate individuals were sampled. Although samples on the same rock platform could potentially be somewhat related (e.g., siblings or parents), sampling was carried out using the same criteria within each population so that, if diversity were underestimated due to relatedness of samples, this artefact should have been consistent across all sites. For *Bostrychia* , sampling involved taking small (< 0.5 cm^2^) pinches of macroalgal tuft from separate clumps on the rock platform. Each sample was wrapped in a fragment of CHUX® Superwipes®, and multiple samples from each site were then placed in 50 ml vials containing 96% ethanol. Examination of individuals from each clade (by G.C. Zuccarello) confirmed their identities as *B* *. intricata* according to morphological criteria. For *Adenocystis*, one thallus was taken from each individual group of thalli, where 'groups' were separated by at least 30 cm; each thallus was squeezed to remove as much internal moisture as possible, and multiple thalli were then placed in 50 ml vials containing 96% ethanol (up to 10 samples per vial). In the laboratory, samples were dried at 60°C for between two and five hours. Desiccated voucher tissue from each sample has been kept in the laboratory, preserved with silica gel.

A small (< 1 mm^2^) fragment of tissue from each sample was isolated using flame-sterilised forceps. DNA extraction followed standard Chelex® procedure (Walsh et al., 1991). Extracted *Adenocystis* DNA was diluted one hundredfold before PCR; this step was not necessary for *Bostrychia* . For each species, a region of mtDNA (partial COI), cpDNA (partial *rbc*L; although in the case of *Adenocystis* the fragment amplified included only the last 198 bases of *rbc*L, followed by 224 bases of intergenic region), and rRNA (partial LSU). PCR amplification was carried out in 20µL volumes containing 1.0µL DNA, 0.5µM of each primer, 1 x buffer, 0.8 mM dNTP, 1.5 mM MgCl_2_, and 1 U Taq polymerase (Bioline ‘BIOTAQ,’ London, UK). Early trials with some relatively ‘universal’ algal primers generally failed or yielded sequences contaminated by non-target DNA, perhaps from epiphytic material; specific primers were therefore developed for each marker for each genus (Table S2), with two exceptions for *Adenocystis*: the primers T01N(F) [24] and T13(R) [25] which successfully amplified a fragment of LSU, and cox 1-789F and cox 1-1378R [26] which sometimes successfully amplified a fragment of COI. Amplification was performed in an Eppendorf Mastercycler® ep gradient using the following profile: 94°C for 2 min; forty cycles of 15s at 94°C, 30s at 50°C, 1 min at 72°C, followed by a final 4 min extension at 72°C. PCR products were purified using an Omega BIO-TEK Ultra-Sep ® Gel Extraction Kit (D2510-02: 500 preps) purification kit – the initial incubation steps were omitted as samples did not require gel purification. DNA fragments were sequenced by the Genetic Analysis Service in the Department of Anatomy and Structural Biology at the University of Otago.

### Analyses

Using Sequencher v. 4.8 (GeneCodes Corporation), sequences were aligned, ambiguous bases assessed / edited, and sequence ends were trimmed to remove primer sequences and ambiguous sections. Extremely high variability towards the 3' end of *Bostrychia* LSU sequences made reliable alignment of this region extremely difficult, so the ambiguous section of the alignment was deleted prior to analysis, and only the first 381 bp of the alignment were used. Dataset parameters were explored using MEGA [27], and uncorrected p distances among lineages were calculated. The transition / transversion bias (R) for each dataset was calculated under the Kimura 2-parameter model [28]. Phylogenies for each DNA data set (individual markers) were constructed using maximum-likelihood (ML) and Bayesian analyses, and included outgroup sequences of related taxa obtained from GenBank (outgroup taxa and accession numbers given in Table S3). In order to roughly compare support for major clades across markers, phylogenies for concatenated data from all markers were also built using ML analyses. The only data set for which a phylogenetic tree was not constructed was the LSU marker of *Adenocystis*, which comprised five unique sequences that provided insufficient phylogenetic information for tree building. The *Adenocystis rbc*L dataset included a published sequence of *Adenocystisutricularis* from St Maxwell Bay in Antarctica (GenBank accession AF385856: Cho et al., 2001), and a published sequence of *Utriculidiumdurvillaei* , a species that is both morphologically and genetically very similar to *A* *. utricularis* (the two are the only genera within the recently erected family Adenocystaceae [29]). ML analyses were performed using PhyML v. 2.4.4 [30] or PhyML v. 3.0 [31] with model parameters as selected by the AICc of jModelTest (see Supplementary Table S4; note that not all models in jModelTest are available for PhyML, and so the closest alternative was chosen where appropriate). Node support was estimated by bootstrapping [32], with heuristic analysis of 1000 replicate data sets. Bayesian posterior probability (PP) values were calculated using MRBAYES 3.1.2 [33] and are shown for the major clades on the ML phylogenies. Markov Chain Monte Carlo (MCMC) searches were performed, with four chains of 10,000,000 generations and trees sampled every 100. The first 10,000 trees sampled were discarded as ‘burn-in,’ as determined using Tracer v 1.4 [34], and a consensus topology and posterior probability values were calculated with the remaining trees. Unrooted statistical parsimony networks were built using TCS 1.21 [35] at the 95% confidence limit.

Population differentiation was assessed using Arlequin population pairwise fixation index (*F*_ST_) values, with significance (*P* < 0.05) based on 1023 random permutations of the data. Populations with only one sample were excluded from these analyses. Arlequin was also used to perform AMOVA analyses on these data sets to assess the relative influence of within- and among-population variance.

Historic population expansions were assessed in the broadly distributed COI clades (clades 1 and 4 for both taxa) by Fu’s neutrality test [36] and mismatch distribution analysis [37], using Arlequin version 3.5 [38]. No other clades were examined as only broad-scale (trans-oceanic) expansions were relevant to the hypotheses and, as the mitochondrial marker was the most informative for each taxon, expansion tests were only carried out on COI data. The fit of the observed to the expected distribution under the sudden-expansion model was assessed with 1000 bootstrap replicates.

All unique DNA sequences obtained during this study were deposited with GenBank (accession numbers JN881465-JN881567) and can be identified by haplotype code. The haplotype frequencies for each site are provided in a separate supporting online Excel table (Table S5).

### Permits

Collection permits for these non-endangered, non-protected seaweed species were not required for sampling in New Zealand and Chile. Collection permits from the sub-Antarctic islands were required, as these vulnerable environmental areas are protected by various administering authorities. For Macquarie Island, a permit for collection of samples was issued by the Department of Primary Industries, Parks, Water and Environment (Tasmania) (permit number 10001), with approval from the Macquarie Island Advisory Group. For Marion Island, a permit for collection was issued by the South African Department of Environmental Affairs (permit reference RES2010/11). For Gough Island, a permit for collection was issued by Tristan da Cunha Conservation. For the Falkland Islands, a collection permit was issued by the Falkland Islands Government (Research license R21/ 2009), and for South Georgia, by the Government of South Georgia (issue date 18 December 2009). For the New Zealand sub-Antarctic islands, entry permits, collection permits, and collection from marine protected areas permits were issued by the Department of Conservation, Southland, New Zealand, in March 2010.

## Results

Each data set comprised sequences of between 400 and 900 bases, with >150 samples amplified for COI, and a subset incorporating representatives from each major COI clade amplified for *rbc*L and LSU (Table 1).

**Table 1:** Data set details for each taxon, indicating the approximate position of the amplified fragment within the gene; the length of the fragment amplified (*note that for *Bostrychia* LSU, only the first 381 bp of the alignment were analysed due to high variability at the 3' end); the number of samples sequenced for each marker (N); the number of variable sites detected; the transition / transversion ratio (*R*); and the number of unique sequences / haplotypes detected.

**Taxon**	**Marker name**	**Marker position**	**Length (bp)**	**N**	**# var. sites**	**Ti / Tv (R)**	**# unique sequences**
*Adenocystis*	COI	centre of gene	485	156	112	2.33	29
*Adenocystis*	'*rbc*L'	198 bp *rbcL* (5'); 224bp intergenic	422	98	31	4.73	16
*Adenocystis*	LSU	centre of gene	900	58	4	1.00	5
*Bostrychia*	COI	centre of gene	401	173	73	7.03	27
*Bostrychia*	*rbc*L	centre of gene	555	63	73	2.27	8
*Bostrychia*	LSU	close to 5' of gene	499-577*	33	18	2.8	8

Numerous highly divergent lineages and clades were resolved in each of the two target species (Figures 1 and 2, and Tables 2 and 3), particularly for the most informative marker, mitochondrial COI. In each case, the chloroplast marker (*rbc*L) was less variable, but also showed several divergent lineages. For both *Adenocystis* and *Bostrychia* , the nuclear marker (LSU) was considerably less informative than the mitochondrial and chloroplast markers. The *Bostrychia* LSU phylogeny comprised several poorly-supported clades, largely concurrent with COI clades with the exception of samples from COI clade 4, which did not form a clade (Figure S4 of the supplementary material). Most *Adenocystis* samples shared a single LSU sequence (sequence 1A in Figure S2). *Adenocystisutricularis* COI clades were monophyletic with respect to all outgroups. For the *A. utricularis* *rbc*L phylogeny, the position of *Utriculidiumdurvillaei* could not be resolved. *Bostrychia* *intricata* was monophyletic with respect to outgroups for COI, consistent with the well-supported branches of the *rbc*L and LSU phylogenies (Figures S3 and S4).

**Table 2:** Genetic distances (uncorrected p distance, %) within and between major clades of *Adenocystis utricularis*.

	**1**	**2**	**3**	**4**
**COI Clade 1**	0.2-0.9			
**COI Clade 2**	4.0-4.7	0.2-0.5		
**COI Clade 3**	6.2-6.7	8.0-8.5	0.5	
**COI Clade 4**	3.2–8.5	5.4–9.6	5.6–9.1	0.3–6.7
	**1**	**2**	**3**	
***rbc*L Clade 1**	0.3-1.1			
***rbc*L Clade 2**	2.5-3.1	0.3		
***rbc*L Clade 3**	2.8-5.1	3.3-4.8	0.3-2.5	
	**1A**	**1B**	**1C**	**1D**
**LSU sequence 1B**	0.1			
**LSU sequence 1C**	0.1	0.2		
**LSU sequence 1D**	0.1	0.2	0.2	
**LSU sequence 1E**	0.1	0.2	0.2	0.2

**Table 3:** Genetic distances (uncorrected p distance, %) within and between major clades / lineages of *Bostrychia intricata*.

	**1**		**2**		**3**				**4**
**COI Clade 1**	0.0-1.3								
**COI Clade 2**	9.6-10.8		0.3						
**COI Clade 3**	7.1-8.4		10.4-12.1		0.3-1.0				
**COI Clade 4**	7.4-8.9		9.5-11.5		2.6-6.0				0.3-2.9
***rbc*L Clade 1**	-								
***rbc*L Clade 2**	6.9		-						
***rbc*L Clade 3**	3.6-4.0		7.0-7.5		0.0-0.6				
***rbc*L Clade 4**	3.6-3.8		7.2-7.4		0.6-1.1				0.2
**LSU Clade 1**	0.0								
**LSU Clade 2**	1.4		0.0						
**LSU Clade 3**	0.6-0.8		0.8-1.1		0.3				
LSU Group* 4	0.3-3.7		1.1-3.7		0.3-3.7				0.3-3.7

The asterisk indicates LSU sequences loosely defined as a group on the basis of the corresponding COI clade for these samples.

Given the high level of genetic divergence observed among clades within each of the study taxa (up to 9% for *A* *. utricularis* COI, and up to 12.1% for *B* *. intricata* COI: Tables 2 and 3), potentially representing cryptic species, interpretations of phylogenetic analyses primarily focused on within-clade patterns; thus, even if any of the major clades represent distinct species, interpretations of patterns of diversity and differentiation should not be confounded by taxonomic issues. Nevertheless, F_ST_ and AMOVA analyses to assess levels of population differentiation were carried out using complete COI data sets, as sample numbers were too low to allow meaningful within-clade population analyses.

**Figure 3:**
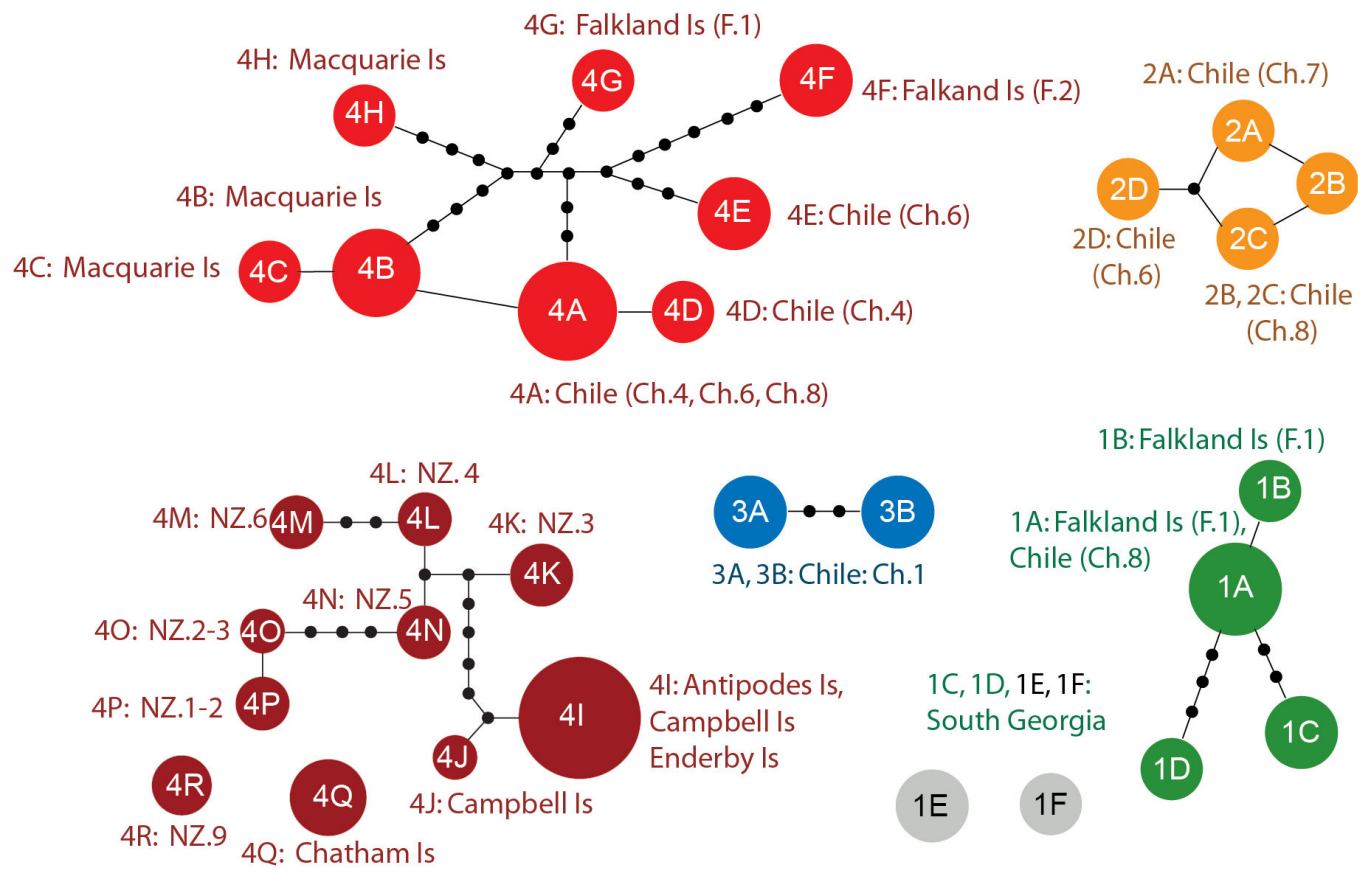
Haplotype networks for *Adenocystisutricularis* mtDNA (COI), illustrating the relationships among haplotypes within clades. Colours correspond to clade colours in Figure 1.

**Figure 4:**
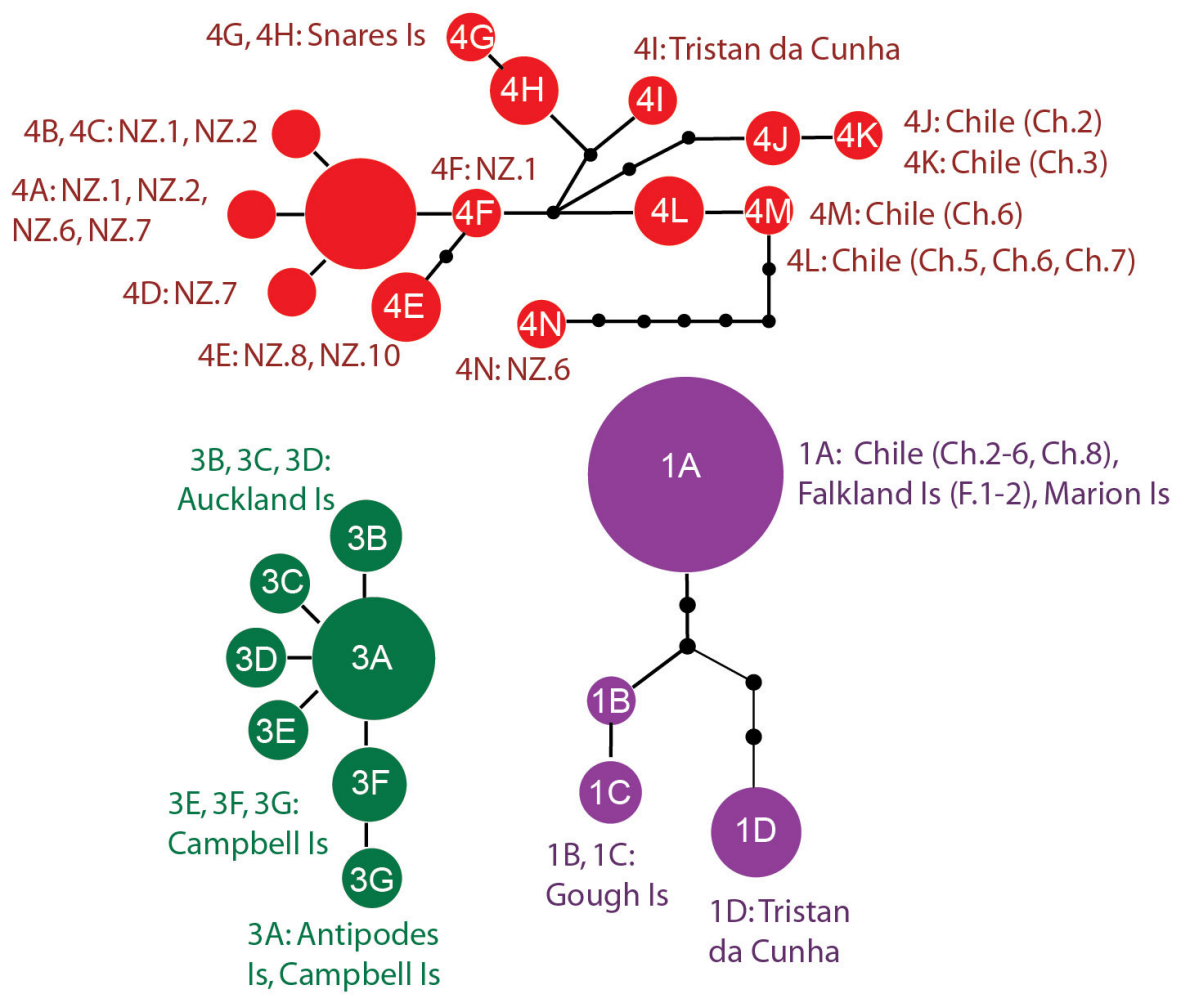
Haplotype networks for *Bostrychia* *intricata* mtDNA (COI), illustrating the relationships among haplotypes within clades. Colours correspond to clade colours in Figure 1.

For both *A* *. utricularis* and *B* *. intricata* , close phylogenetic relationships – in some cases even shared haplotypes – were observed among populations separated by large oceanic distances. For example, some COI *A* *. utricularis* haplotypes from Macquarie Island (haplotypes 4B and 4C) differed only by one to two base pairs (0.3-0.5%) from haplotypes detected in southern Chile (haplotypes 4A and 4D), and haplotype 1A in *A* *. utricularis* COI clade 1 was shared by populations in southern Chile and the Falkland Islands (Figures 1 and 3). Close relationships were also detected between South Georgian and Chile / Falkland Island haplotypes (1A and 1B *vs*1C-1F: 0.3-0.6% distant). Similarly close relationships among New Zealand, Chilean and Falkland Island *A* *. utricularis* were observed for the chloroplast marker (e.g., as low as 0.3% between haplotypes 3A or 3C in New Zealand and haplotypes 3I from the Falkland Islands or 3J from Chile and the Falkland Islands, respectively: see clade 3 in Figure S1 of the supplementary material), and almost no variation was observed across vast distances in the ribosomal marker (maximum 0.2%: Table 2; and see Figure S2 of supplementary material). For *B* *. intricata* , one haplotype (COI: haplotype 1A, Figures 2 and 4; *rbc*L: haplotype 1A, Figure S3) was shared among extremely distant sites on Marion Island, the Falkland Islands, and throughout southern Chile. Within COI clade 4 some New Zealand haplotypes were more closely related to some Chilean haplotypes than they were to other New Zealand haplotypes (e.g., for COI, 4F is more closely related to 4L (0.5%) than to 4N (2.1%): see Figure 2; and for *rbc*L, haplotype 4A was shared among New Zealand and Chilean sites: see Figure S3 of the supplementary material).

The population pairwise *F*_ST_ analyses (Supplementary tables S6 and S7) indicate that some disjunct populations were genetically similar (note that, as sample sizes at several populations were low, these results should be interpreted cautiously). For example, for *B* *. intricata* , Falkland Island and several Chilean populations were not significantly differentiated. Nevertheless, in some populations several divergent clades were present, which, combined with low sample sizes, appears to have led to the failure of many of the trans-oceanic genetic similarities noted in the preceding paragraph to be reflected in the populations pairwise *F*_ST_ comparisons. All AMOVAs were highly significant, and showed the vast proportion of genetic variation to be among rather than within populations (Table 4).

**Table 4:** AMOVA of within- and among-population differences for COI data sets.

**Taxon**		**d.f.**	**S.S.**	**% variation**	***P***
*Adenocystisutricularis*	Among	19	1792.1	90.53	0.000
*Adenocystisutricularis*	Within	129	169.4	9.47	
*Bostrychia* *intricata*	Among	20	1303.2	80.07	0.000
*Bostrychia* *intricata*	Within	154	293.8	19	

## Discussion

Despite clear geographic partitioning of genetic lineages (i.e. some clades being confined to particular geographic regions), both *A* *. utricularis* and *B* *. intricata* also showed unequivocal evidence of long-distance, trans-oceanic dispersal. Although our genetic data indicate the possibility of cryptic species within both taxa (see below) the patterns of genetic diversity observed nonetheless allow assessment of the trans-oceanic dispersal hypothesis. For example, *A* *. utricularis* showed low genetic distances among the Falkland Islands, South Georgia and southern Chile, as well as across ~ 8000 km of the Pacific Ocean (Figure 1), and throughout the NZ subantarctic. Similarly, *B* *. intricata* COI exhibited evidence of dispersal throughout the NZ subantarctic, and between NZ and Chile (Figure 2), and a single *B* *. intricata* COI haplotype (1A) was shared by populations in Chile, the Falkland Islands, and Marion Island, implying dispersal of reproductively viable adult thalli across approximately 6,500 km of open ocean. Future research on whether all lineages – including those that are appear most ancient and have restricted distributions – are equally capable of dispersal (e.g., whether all can grow on floating substrata such as wood or kelp), would help to shed light on the evolution of dispersal mechanisms.

Despite the evidence that long-distance dispersal is possible for both *B* *. intricata* and *A* *. utricularis* , the maintenance of genetic diversity on small spatial scales suggests that it might only rarely result in gene flow or establishment of emigrants’ offspring. Research is increasingly indicating that density-dependent processes play a critical role in driving spatial genetic and ecological structure, with founding colonizers of disturbed or new habitat rapidly reaching high densities, preventing establishment of latecomers (‘founder takes all’ [39]). Thus, for many species, dispersal events may occur frequently but primarily permit opportunistic colonisation of newly-available habitat, rather than facilitating ongoing long-distance genetic connectivity (but see 7). Future research should aim to directly test whether such a process is indeed responsible for spatial genetic structure in these intertidal algal species.

Many macroalgal species may have some level of (usually short-lived) buoyancy, if only due to small air bubbles trapped around their fronds. Mature *A* *. utricularis* thalli consist of small sacs filled with liquid [23] and are only poorly buoyant (C. Fraser pers*. obs.*). In addition, they are considerably less robust than the large, tough kelps (e.g., *Durvillaea* and *Macrocystis*), and it is unlikely that they could survive long voyages floating at the sea surface. Nonetheless, the evidence from this study suggests that *A* *. utricularis* can indeed travel vast, trans-oceanic distances. *Adenocystisutricularis* grows in the low intertidal, and shallow subtidal, and it is possible (although as-yet unobserved) that adult thalli, or gametophytic filaments, can grow on - or entwine with - holdfasts of the large kelps, and are able to raft across oceans on these more-buoyant hosts.

High genetic diversity was detected within the New Zealand *A* *. utricularis* COI group 4I-4R (up to 7.3% for COI: Table 2), with samples from populations north of the Canterbury Bight being distinct from all more southern New Zealand populations. That the Canterbury Bight – a 200 km-long unbroken stretch of cobbled beach south of Banks Peninsula – forms a significant biogeographic barrier for rocky-shore marine macroalgal taxa has previously been demonstrated in two bull-kelp (*Durvillaea*) species [40,41], the chiton *Sypharochitonpelliserpentis* [42] and the gastropod *Diloma* *arida* [43].

Recently, patterns of genetic connectivity between New Zealand and South America have been observed in another non-buoyant, intertidal macroalga that was previously hypothesised to be a poor disperser. Boedeker et al. [44] noted close genetic (nuclear ITS) similarities among populations of the turfing green alga *Wittrockiella* *lyalli* on either side of the South Pacific Ocean. The authors suggested this alga may be able to traverse oceans by rafting on buoyant wood, as the species has been observed growing on tree logs in the intertidal zone. Similarly, during this study we observed *B* *. intricata* growing on detached driftwood in the intertidal zone, and it seems probable that this species is able to disperse using that buoyant substratum as a raft. Anthropogenic means of dispersal such as hull fouling or ballast water of ships [45] could also potentially allow both species to traverse oceans; although we could find no record of either species in a ship fouling or ballast environment, such dispersal cannot be categorically ruled out. Future work could aim to assess broad-scale phylogeographic patterns in these species in relation to major shipping routes. Nevertheless, it seems unlikely that the very short-lived gametes of either species (see Introduction) could survive an ocean traverse in ship ballast, and these macroalgae would be unlikely to be tolerated on ship hulls to the age and size at which they could release gametes.

Molecular studies are increasingly indicating that long-distance dispersal of both buoyant and non-buoyant species of macroalgae is possible (e.g. see 14,15,18,46), although some species and geographic regions show greater evidence of trans-oceanic dispersal than others [46]. Rafting, in particular, appears to have played a critical role in facilitating long-distance dispersal and structuring intertidal ecosystems [13]. Many species that lack obvious trans-oceanic dispersal ability (e.g., brooding invertebrates [4,5,7]:; small, non-buoyant macroalgae [44]: and this study; and even terrestrial vertebrates [47]:) are nonetheless evidently capable of capitalising on the buoyancy of floating wood or detached kelp to disperse long distances. The long-distance, trans-oceanic dispersal events inferred for both *A* *. utricularis* and *B* *. intricata* were most probably facilitated by the strong Antarctic Circumpolar Current, which has previously been implicated in dispersal of numerous taxa around the high-latitudes of the Southern Hemisphere (see references in [13]). Long-distance dispersal of macroalgae naturally relies on effective dispersal means and vectors (such as rafting on buoyant material in strong ocean currents), and its likelihood for any species or region must therefore be considered on an entirely case-by-case basis.

We observed considerable genetic diversity within and among populations of both *B* *. intricata* and *A* *. utricularis* in regions that are thought to have been affected by ice at the Last Glacial Maximum (high-latitude subantarctic islands and Chilean fiordland; see 14,41,48). Under a postglacial recolonisation hypothesis, populations of taxa that were driven locally extinct in glaciated regions, but have since recolonised these areas, are expected to show considerably lower genetic diversity in such regions than elsewhere [48,49]. In contrast to ice-scour susceptible kelps such as *Durvillaeaantarctica* and *Macrocystispyrifera* , which have been found to show negligible genetic diversity at high latitudes [14,15,41], the diversity observed in such regions for both *B* *. intricata* and *A* *. utricularis* suggests that these species may have persisted in some ice-affected regions throughout the LGM. Such a hypothesis is consistent with the biology of these macroalgae; *D* *. antarctica* and *M* *. pyrifera* are apparently unable to survive in ice-affected locations today [50], whereas *B* *. intricata* and *A* *. utricularis* can. *Bostrychia* *intricata* is recorded from contemporarily sea-ice affected regions, including Antarctica [51], and can survive several days of freezing [52]. *Adenocystisutricularis* has a circum-subantarctic distribution and occurs in ice-affected regions such as the South Shetland Islands [53], presumably due to its persistence as a filamentous gametophyte during winter when ice is most extensive.

### Taxonomic issues

A key difficulty in undertaking broad-scale phylogeographic analyses in groups that have had only limited attention from molecular biologists is that cryptic species may confound both sampling strategies and data analyses. Macroalgal morphology is highly plastic, and molecular studies are revealing the need for taxonomic revision of many macroalgal groups traditionally classified by morphological and life history traits alone (e.g., [54–57]). The need for further research to verify taxonomic issues within the red algal genus *Bostrychia* has been highlighted by previous molecular work revealing that some species are polyphyletic [58], by physiological studies suggesting some differences in temperature tolerance within *B* *. intricata* from different locations [21,52], and by previous molecular work on *B* *. intricata* indicating possible cryptic species [59]. Our results indicate that there are at least four highly divergent (2.4-10.8% uncorrected p distance for COI) clades within *Bostrychia* *intricata* around the high latitudes of the Southern Hemisphere. No obvious morphological differences among phylogenetic clades were observed. Although some clades were restricted to distinct geographic regions (e.g., COI clade 3 in the New Zealand subantarctic), others are more broadly distributed and have overlapping ranges, with multiple lineages detected within sites (e.g., COI clades 1 and 4). That these lineages can be so genetically divergent even in sympatry suggests that they may represent distinct species. Only a subset of samples were analysed using the nuclear marker (LSU), so detailed analysis of which sympatric lineages are reproductively isolated is not possible here; nevertheless, the presence of two divergent LSU lineages of *B* *. intricata* at Wellington (site NZ.1) and at one site in southern Chile (Ch. 6) (Figure S4 in the supplementary material) supports the notion that the sampling may have included several as-yet unrecognised species. Further taxonomic research is clearly required to elucidate phylogenetic relationships and species boundaries within *Bostrychia* , including samples from a wider range of locations. In particular, future research on *B* *. intricata* should assess the taxonomic status of different genetic lineages found in high-latitude Southern Hemisphere locations (this study and [59]) and in lower-latitude populations in Australia and South Africa [59].

*Adenocystis* has been considered a monospecific genus; at the time of most sample collection for this study (2010-2011), only *A* *. utricularis* was listed as taxonomically valid [60]. Our phylogeographic research revealed several divergent lineages within *A* *. utricularis* , with the level of inter-lineage genetic divergence (COI) for *A* *. utricularis* (3.2-9.6%) comparable to inter-specific distances observed in numerous other brown algal taxa [61]. There is therefore a strong possibility that the samples of *A* *. utricularis* that we collected comprise numerous cryptic species. A morphological study by Asensi et al. [62] proposed an additional species from southern Chile, *A* *. longissima* [62]. A published LSU sequence of *A* *. longissima* (GenBank accession AF071779) did not match any of the LSU sequences obtained during this study (see Figure S2), but we detected very little variation within LSU overall in this genus, and whether the taxon erected by Asensi et al. [62] corresponds to any of the lineages detected by our study is not clear. Future genetic and morphological work should certainly aim to resolve taxonomic issues in this genus. Most genetic diversity within *A* *. utricularis* was detected in the South American region, with four of the five clades occurring in southern Chile. Southern South America may therefore be a centre for radiation in this genus, perhaps partly due to the environmental heterogeneity of the extensive and complex Chilean fjords.

## Supporting Information

Figure S1 Phylogeographic relationships within *Adenocystisutricularis* based on *rbc*L data. The phylogenetic tree (lower right corner) indicates relationships among, and diversity within, major clades; support for these clades is shown by Bayesian Posterior Probability values above branches, and ML bootstraps below. Most outgroup taxa have been removed for clarity. The maps indicate clade distributions and proportions at each locality, with multiple haplotypes at a site indicated by pie divisions. Haplotype networks (95% confidence limit) indicate the relationships among haplotypes within clades, with their distribution among landmasses indicated approximately by their positions across regional divisions (dashed lines).
(TIF)

Figure S2 Phylogeographic relationships within *Adenocystisutricularis* based on LSU data, indicated by a haplotype network (95% confidence limit). The maps indicate sequence distributions and proportions at each locality, with multiple sequences at a site indicated by pie divisions. Published sequences of the outgroup taxon *Utriculidiumdurvillei* and the proposed species *A* *. utricularis* *f*. *longissima* are included.
(TIF)

Figure S3 Phylogeographic relationships within *Bostrychia* *intricata* based on *rbc*L data. The phylogenetic tree (lower right corner) indicates relationships among, and diversity within, major clades; support for these clades is shown by Bayesian Posterior Probability values above branches, and ML bootstraps below. Most outgroup taxa have been removed for clarity. The maps indicate clade distributions and proportions at each locality, with multiple haplotypes at a site indicated by pie divisions. Haplotype networks (95% confidence limit) indicate the relationships among haplotypes within clades, with their distribution among landmasses indicated approximately by their positions across regional divisions (dashed lines); note the exception of Tristan da Cunha.
(TIF)

Figure S4 Phylogeographic relationships within *Bostrychia* *intricata* based on LSU data. Node support is shown by Bayesian Posterior Probability values above branches, and ML bootstraps below. Outgroup taxa have been removed for clarity. Red stars indicate groups that share closely-related or identical sequences across vast (trans-oceanic) distances.
(TIF)

Table S1 Collection locations. Most co-ordinates estimated using Google, Earth.
(DOCX)

Table S2 Primer pairs used in PCR amplification. (DOCX)

Table S3 Outgroups used in phylogenetic analyses. (DOCX)

Table S4 Model parameters implemented in PhyML analyses. Models are based on parameters recommended by the AICc of jModeltest, although GTR models replaced some more complex models (e.g., TIM3) that are not available in PhyML.
(DOCX)

Table S5 Haplotype frequencies by site for each marker and each taxon. Haplotypes are identified by haplotype code (e.g., 1A).
(DOCX)

Table S6 Population pairwise F_ST_ for *A* *. utricularis* COI (**excluding where n=1**). Cells marked with an asterisk were non-significant (P > 0.05).
(XLS)

Table S7 Population pairwise F_ST_ for *B* *. intricata* COI (excluding where n=1). Cells marked with an asterisk were non-significant (P > 0.05).
(XLS)
