# Supplementary figures and images for: Genetic Affinities between Trans-Oceanic Populations of Non-Buoyant Macroalgae in the High Latitudes of the Southern Hemisphere
Source: PLoS One. 2013 Jul 22;8(7):e69138. doi: 10.1371/journal.pone.0069138 (PMC3718832; doi:10.1371/journal.pone.0069138)

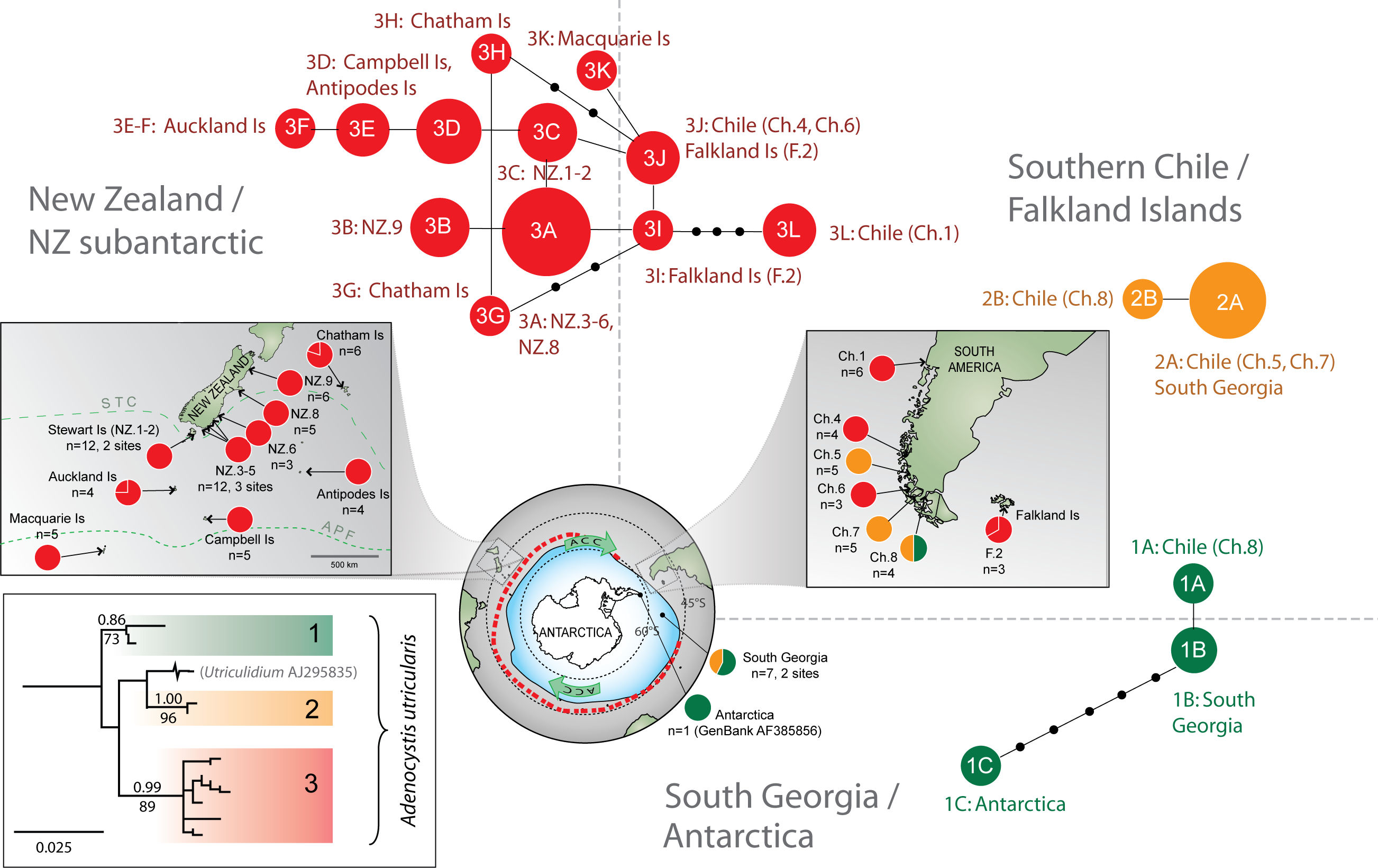

Supplement: Figure S1 — The phylogenetic tree (lower right corner) indicates relationships among, and diversity within, major clades; support for these clades is shown by Bayesian Posterior Probability values above branches, and ML bootstraps below. Most outgroup taxa have been removed for clarity. The maps indicate clade distributions and proportions at each locality, with multiple haplotypes at a site indicated by pie divisions. Haplotype networks (95% confidence limit) indicate the relationships among haplotypes within clades, with their distribution among landmasses indicated approximately by their positions across regional divisions (dashed lines). (TIF) [file pone.0069138.s001.tif]

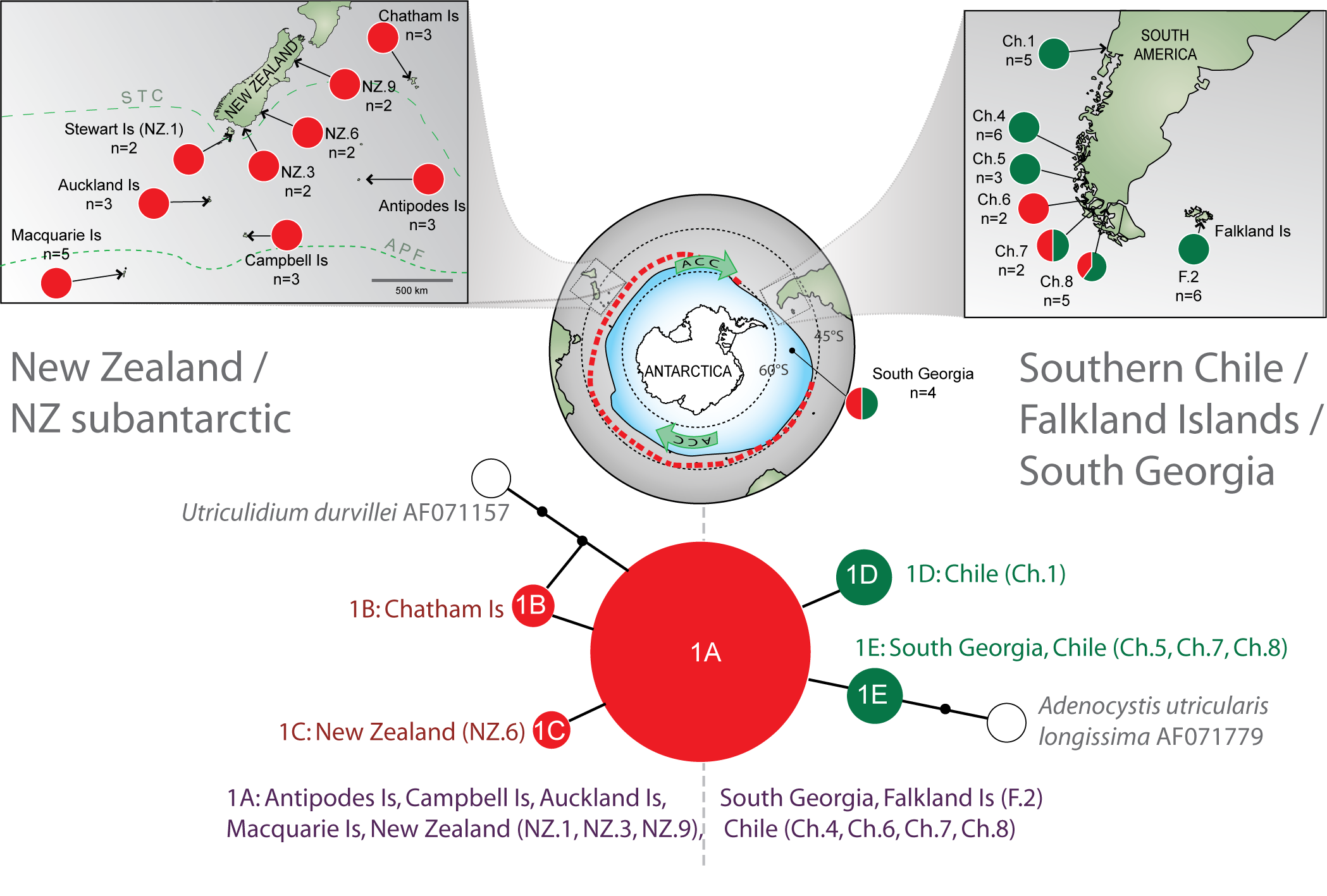

Supplement: Figure S2 — The maps indicate sequence distributions and proportions at each locality, with multiple sequences at a site indicated by pie divisions. Published sequences of the outgroup taxon Utriculidiumdurvillei and the proposed species A . utricularis f. longissima are included. (TIF) [file pone.0069138.s002.tif]

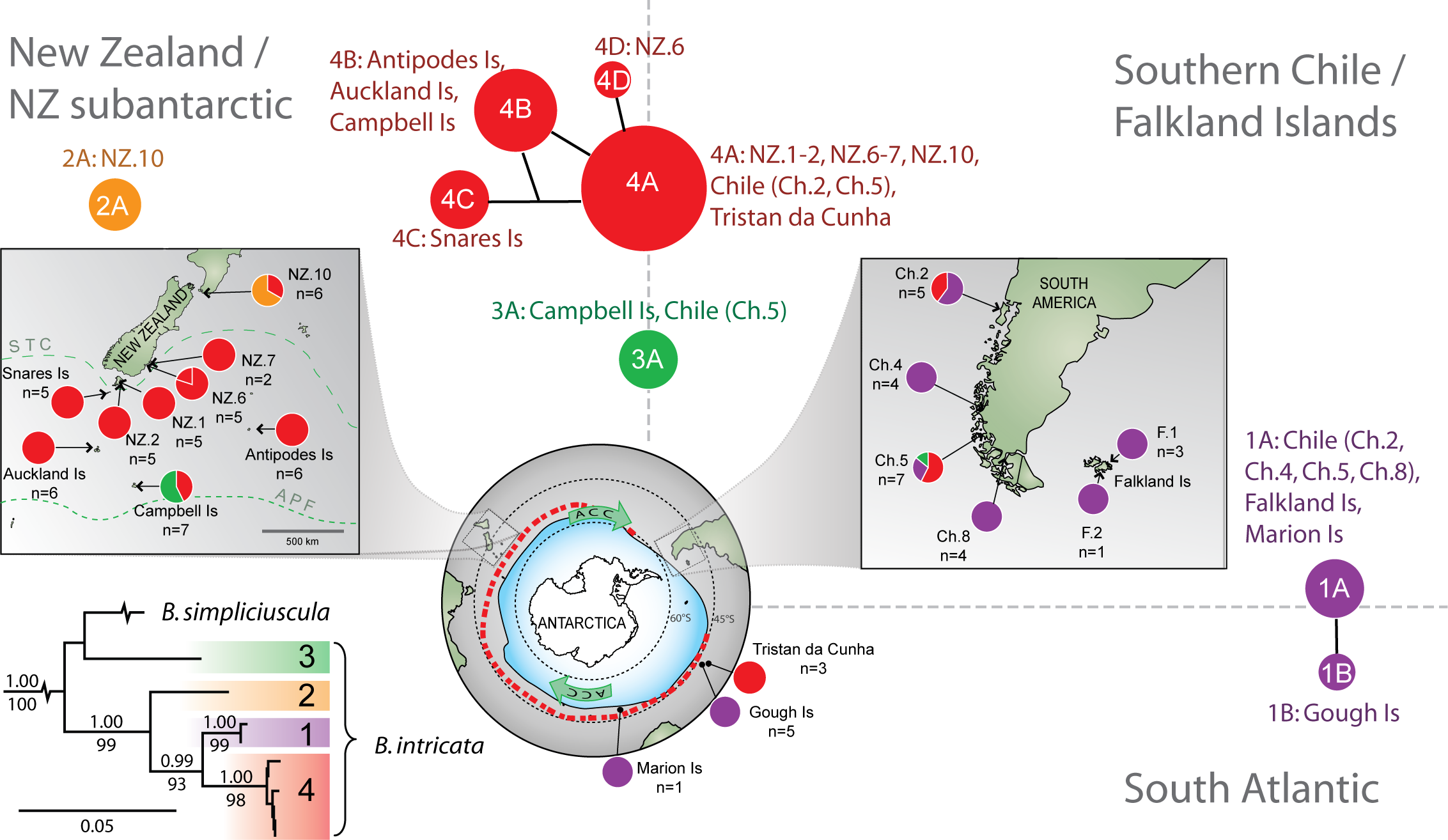

Supplement: Figure S3 — The phylogenetic tree (lower right corner) indicates relationships among, and diversity within, major clades; support for these clades is shown by Bayesian Posterior Probability values above branches, and ML bootstraps below. Most outgroup taxa have been removed for clarity. The maps indicate clade distributions and proportions at each locality, with multiple haplotypes at a site indicated by pie divisions. Haplotype networks (95% confidence limit) indicate the relationships among haplotypes within clades, with their distribution among landmasses indicated approximately by their positions across regional divisions (dashed lines); note the exception of Tristan da Cunha. (TIF) [file pone.0069138.s003.tif]

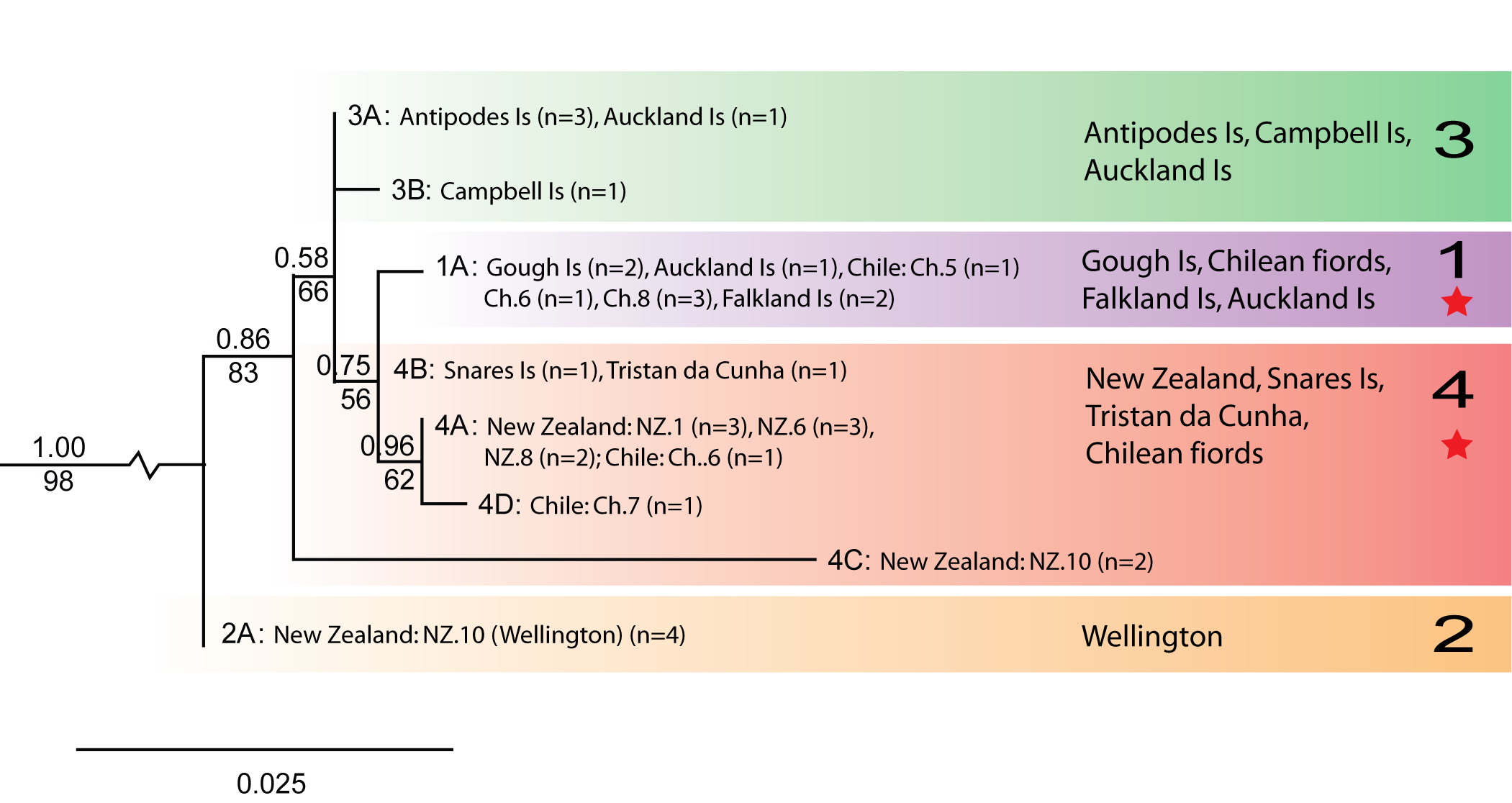

Supplement: Figure S4 — Node support is shown by Bayesian Posterior Probability values above branches, and ML bootstraps below. Outgroup taxa have been removed for clarity. Red stars indicate groups that share closely-related or identical sequences across vast (trans-oceanic) distances. (TIF) [file pone.0069138.s004.tif]
