# Supplementary material for: Genetic Affinities between Trans-Oceanic Populations of Non-Buoyant Macroalgae in the High Latitudes of the Southern Hemisphere
Source: PLoS One. 2013 Jul 22;8(7):e69138. doi: 10.1371/journal.pone.0069138 (PMC3718832; doi:10.1371/journal.pone.0069138)
Supplement: Table S1 — Most co-ordinates estimated using Google, Earth. (DOCX) [file pone.0069138.s005.docx]

| **Region** | **Site code** | **Site name** | **Latitude** | **Longitude** |
| --- | --- | --- | --- | --- |
| New Zealand | NZ.1 | Stewart Island: Ringaringa | 46°54'9.07”S | 168° 8'41.10”E |
| New Zealand | NZ.2 | Stewart Island: The Neck | 46°55'25.37”S | 168°11'26.95”E |
| New Zealand | NZ.3 | Waipapa Point | 46°39'43.47”S | 168°50'47.49”E |
| New Zealand | NZ.4 | Curio Bay | 46°39'46.15”S | 169° 6'3.88”E |
| New Zealand | NZ.5 | Akatore | 46° 6'46.05”S | 170°11'35.94”E |
| New Zealand | NZ.6 | Brighton | 45°56'54.91”S | 170°20'12.72”E |
| New Zealand | NZ.7 | Doctors Point | 45°44'9.38”S | 170°36'59.98”E |
| New Zealand | NZ.8 | Banks Peninsula: Te Oka Bay | 43°51'23.79”S | 172°46'14.44”E |
| New Zealand | NZ.9 | Kaikoura | 42°25'18.00”S | 173°43'21.12”E |
| New Zealand | NZ.10 | Wellington: Moa Point | 41°20'44.32”S | 174°48'31.97”E |
| Chile | Ch.1 | Cochulgue | 36°35'38.47”S | 72°58'42.33”W |
| Chile | Ch.2 | Punihuil | 41°55'45.88”S | 74° 1'59.53”W |
| Chile | Ch.3 | Puerto Montt | 41°28'23.82”S | 72°56'27.56”W |
| Chile | Ch.4 | CS7 | 50º28'16.4”S | 74º16'50.3”W |
| Chile | Ch.5 | CS4 | 51º09'50.1”S | 73º42'52.9”W |
| Chile | Ch.6 | CS2 | 51º46'43.7”S | 73º43'00.6” W |
| Chile | Ch.7 | CS1 | 52º15'36”S | 73º41'04” W |
| Chile | Ch.8 | Punta Arenas | 53º37'34.0”S | 70º55'12.7” W |
| Oceanic Islands | F.1 | Falklands: Cape Pembroke | 51°40'57.13”S | 57°43'2.44”W |
| Oceanic Islands | F.2 | Falklands: Sea Lion Island | 52°25'45.60”S | 59° 3'6.53”W |
| Oceanic Islands | N/A | Macquarie Island | 54°29'57.18”S | 158°56'34.50”E |
| Oceanic Islands | N/A | Marion Island | 46°52'36.03”S | 37°51'39.47”E |
| Oceanic Islands | N/A | South Georgia: Grytviken | 54°15'10.79”S | 36°28'19.25”W |
| Oceanic Islands | N/A | South Georgia: Cobblers Cove | 54°16'36.47”S | 36°17'59.32”W |
| Oceanic Islands | N/A | Gough Island | 40°21'7.86”S" | 9°52'58.02”W |
| Oceanic Islands | N/A | Tristan da Cunha | 37° 3'50.21”S | 12°18'52.41”W |
| Oceanic Islands | N/A | Campbell Island | 52°31'0.90”S | 169°11'51.49”E |
| Oceanic Islands | N/A | Auckland Islands: Enderby Is | 50°30'19.89”S | 166°17'27.39”E |
| Oceanic Islands | N/A | Snares Islands | 48° 0'27.47”S | 166°32'54.62”E |
| Oceanic Islands | N/A | Antipodes Islands | 49°41'29.93”S | 178°48'46.35”E |
| Oceanic Islands | N/A | Chatham Islands | 43°48'39.91”S | 176°40'26.46”W |
