# Supplementary material for: Genetic Affinities between Trans-Oceanic Populations of Non-Buoyant Macroalgae in the High Latitudes of the Southern Hemisphere
Source: PLoS One. 2013 Jul 22;8(7):e69138. doi: 10.1371/journal.pone.0069138 (PMC3718832; doi:10.1371/journal.pone.0069138)
Supplement: Table S2 — (DOCX) [file pone.0069138.s006.docx]

| **Primer (dir)** | **Primer sequence** | **Target** | **Source** |
| --- | --- | --- | --- |
| BstCF2 (F) | 5’ GATTTTTTTTATGGTTATGCC | COI | This study |
| BstCR2 (R) | 5’ GTATTRAARTTTCTATCAGTAAG | COI | This study |
| BstRF1 (F) | 5’ CCATTTATGCGTTGGAA | *rbc*L | This study |
| BstRR4 (R) | 5’ GATTTGCTGTTGCTCCTG | *rbc*L | This study |
| BstRF2 (F) | 5’ CAAYTCACAACCATTTATG | *rbc*L | This study |
| BstRR5 (R) | 5’ GCTCCTGCYTGAATWCCATC | *rbc*L | This study |
| BstLSUF1 (F) | 5’ YAGTAGCGGCGAGCGAAGAG | LSU | This study |
| BstLSUR1 (R) | 5’ CAAGCATAGTTCACCATCTTTC | LSU | This study |
| AdCF1 (F) | 5’ GGTTTTGGAATYGTTAGTC | COI | This study |
| AdCF2 (F) | 5’ GTCATATTCTTTCTACTCTTGG | COI | This study |
| AdCR1 (R) | 5’ CCCAGGARGTGCATCGG | COI | This study |
| Cox1-789F (F) | 5’ TNTAYCARCATTTATTTTGGTT | COI | Silberfeld *et al.*, 2010 |
| Cox1-1378R (R) | 5’ TCYGGNATACGNCGNGGCATACC | COI | Silberfeld *et al.*, 2010 |
| AdRF1 (F) | 5’ TACTATTGGTCATCCAGATGG | *rbc*L | This study |
| AdRR1 (R) | 5’ TTAGACATAGCATAACCAACTTG | *rbc*L | This study |
| T01N (F) | 5’ GATGACCCGCTGAATTTAAG | LSU | Harper & Saunders, 2001b |
| T13 (R) | 5’ GCAGGTGAGTTGTTACACTC | LSU | Harper & Saunders, 2001a |
