# Supplementary material for: Genetic Affinities between Trans-Oceanic Populations of Non-Buoyant Macroalgae in the High Latitudes of the Southern Hemisphere
Source: PLoS One. 2013 Jul 22;8(7):e69138. doi: 10.1371/journal.pone.0069138 (PMC3718832; doi:10.1371/journal.pone.0069138)
Supplement: Table S3 — (DOCX) [file pone.0069138.s007.docx]

| **Ingroup genus** | **Gene** | **Outgroup name** | **Genbank accession** |
| --- | --- | --- | --- |
| *Adenocystis* | COI | *Ectocarpus* sp. | AF037994 |
| *Adenocystis* | COI | *Saccharina* *longipedalis* | AP011497 |
| *Adenocystis* | COI | *Tilopteris* *mertensii* | EU681430 |
| *Adenocystis* | COI | *Splachnidium rugosum* | EU681427 |
| *Adenocystis* | COI | *Scytothamnus australis* | EU681425 |
| *Adenocystis* | *rbc*L | *Analipus* *japonicus* | AY095323 |
| *Adenocystis* | *rbc*L | *Caepidium* *antarcticum* | AJ295826 |
| *Adenocystis* | *rbc*L | *Hincksia* *mitchelliae* | U38753 |
| *Adenocystis* | *rbc*L | *Kuckuckia* sp. | U38703 |
| *Adenocystis* | *rbc*L | *Petalonia* *binghamiae* | AF385843 |
| *Adenocystis* | *rbc*L | *Scytosiphon* *lomentaria* | AF207811 |
| *Adenocystis* | *rbc*L | *Utriculidium* *durvillei* | AJ295835 |
| *Adenocystis* | LSU | *Laminaria* *saccharina* | AY441782 |
| *Adenocystis* | LSU | *Scytosiphon* *lomentaria* | D16558 |
| *Adenocystis* | LSU | *Utriculidium* *durvillei* | AF071157 |
| *Adenocystis* | LSU | *Utriculidium* *durvillei* | AJ295821 |
| *Adenocystis* | LSU | *Ectocarpus* *crouaniorum* | FN564442 |
| *Bostrychia* | COI | *Polysiphonia* sp. | GU385829 |
| *Bostrychia* | COI | *Laurencia* sp. | GU223891 |
| *Bostrychia* | *rbc*L | *Acrosorium* *polyneurum* | AF254153 |
| *Bostrychia* | *rbc*L | *Cryptopleura* *callophylloides* | AF254172 |
| *Bostrychia* | *rbc*L | *Bostrychia* *simpliciuscula* | AY920827 |
| *Bostrychia* | LSU | *Ptilota* *serrata* | EF033605 |
| *Bostrychia* | LSU | *Sorella* *repens* | EF033608 |
| *Bostrychia* | LSU | *Gelidium serrulatum* | AF039538 |
