# Supplementary material for: Genetic Affinities between Trans-Oceanic Populations of Non-Buoyant Macroalgae in the High Latitudes of the Southern Hemisphere
Source: PLoS One. 2013 Jul 22;8(7):e69138. doi: 10.1371/journal.pone.0069138 (PMC3718832; doi:10.1371/journal.pone.0069138)
Supplement: Table S4 — Models are based on parameters recommended by the AICc of jModeltest, although GTR models replaced some more complex models (e.g., TIM3) that are not available in PhyML. (DOCX) [file pone.0069138.s008.docx]

|  | ***Adenocystis*** | | | | ***Bostrychia*** | | | |
| --- | --- | --- | --- | --- | --- | --- | --- | --- |
|  | **COI** | ***rbc*L** | **LSU** | **Concatenated** | **COI** | ***rbc*L** | **LSU** | **Concatenated** |
| **Model** | GTR + G | TN + G | N/A | GTR + I + G | HKY + G | GTR + G | GTR + I + G | GTR + I + G |
| **Pinv** | N/A | N/A | N/A | 0.295 | N/A | N/A | 0.235 | 0.329 |
| **Gamma shape** | 0.192 | 0.596 | N/A | 0.236 | 0.162 | 0.193 | 1.165 | 0.018 |
| **Tree improvement** | SPR | SPR | N/A | SPR | SPR | SPR | SPR | SPR |
