# Supplementary material for: Genetic Affinities between Trans-Oceanic Populations of Non-Buoyant Macroalgae in the High Latitudes of the Southern Hemisphere
Source: PLoS One. 2013 Jul 22;8(7):e69138. doi: 10.1371/journal.pone.0069138 (PMC3718832; doi:10.1371/journal.pone.0069138)
Supplement: Table S5 — Haplotypes are identified by haplotype code (e.g., 1A). (DOCX) [file pone.0069138.s009.docx]

|  | ***Adenocystis*** | | | ***Bostrychia*** | | |
| --- | --- | --- | --- | --- | --- | --- |
| **Site name** | **COI** | ***rbc*L** | **LSU** | **COI** | ***rbc*L** | **LSU** |
| NZ.1: Stewart Island: the Neck | 4Px7;4Ox3 | 3Cx6 | 1Ax2 | 4Ax6;4Bx1;4Fx1 | 4Ax5 | 4Ax3 |
| NZ.2: Stewart Island: Ringaringa | 4Px7 | 3Cx6 |  | 4Ax2;4Bx1;4Cx3;4Fx1 | 4Ax5 |  |
| NZ.3: Waipapa Point | 4Kx7;4Ox1 | 3Ax6 | 1Ax2 |  |  |  |
| NZ.4: Curio Bay | 4Lx5 | 3Ax2 |  |  |  |  |
| NZ.5: Akatore | 4Nx5 | 3Ax4 |  |  |  |  |
| NZ.6: Brighton | 4Mx6 | 3Ax3 | 1Cx2 | 4Ax8;4Nx1 | 4Ax4;4Dx1 | 4Ax3 |
| NZ.7: Doctors Point |  |  |  | 4Ax3;4Dx1 | 4Ax2 |  |
| NZ.8: Banks Peninsula: Te Oka Bay |  | 3Ax5 |  | 4Ex8 |  | 4Ax2 |
| NZ.9: Kaikoura | 4Qx10 | 3Bx6 | 1Ax2 |  |  |  |
| NZ.10: Wellington: Moa Point |  |  |  | 4Ex2;2Ax6;2Bx1 | 2Ax4;4Ax2 | 2Ax4;4Cx2 |
| Chile: Ch.1: Cochulgue | 3Ax5;3Bx3 | 3Lx6 | 1Dx5 |  |  |  |
| Chile: Ch.2: Punihuil |  |  |  | 1Ax4;4Jx2 | 1Ax3;4Ax2 |  |
| Chile: Ch.3: Puerto Montt |  |  |  | 1Ax6;4Kx1 |  |  |
| Chile: Ch.4: Cruise Site 7 | 4Ax7;4Dx1 | 3Jx4 | 1Ax6 | 1Ax8 | 1Ax4 |  |
| Chile: Ch.5: Cruise Site 4 |  | 2Ax5 | 1Ex3 | 1Ax1;4Lx3 | 1Ax2;3Ax1;4Ax4 | 1Ax1 |
| Chile: Ch.6: Cruise Site 2 | 2Dx1;4Ax3;4Ex6 | 3Jx3 | 1Ax2 | 1Ax1;4Lx2;4Mx3 |  | 1Ax1;4Ax1 |
| Chile: Ch.7: Cruise Site 1 | 2Ax1 | 2Ax5 | 1Ax1;1Ex1 | 4Lx5 |  | 4Dx1 |
| Chile: Ch.8: Punta Arenas | 1Ax2;2Bx3;2Cx1;4Ax1 | 1Ax2;2Bx2 | 1Ax2;1Ex3 |  | 1Ax4 | 1Ax3 |
| Falklands: Cape Pembroke | 1Ax7;1Bx2;4Gx1 |  |  | 1Ax14 | 1Ax3 | 1Ax2 |
| Falklands: Sea Lion Island | 4Fx5 | 3Ix2;3Jx1 | 1Ax6 | 1Ax9 | 1Ax1 |  |
| Macquarie Island | 4Bx8;4Cx1;4Hx1 | 3Kx5 | 1Ax6 |  |  |  |
| Marion Island |  |  |  | 1Ax1 | 1Ax1 |  |
| South Georgia: Grytviken | 1Ex6;1Fx1 | 1Bx4 | 1Ax2;1Ex2 |  |  |  |
| South Georgia: Cobblers Cove | 1Cx7;1Dx1 | 1Bx4 |  |  |  |  |
| Gough Island |  |  |  | 1Bx7;1Cx4 | 1Bx5 | 1Ax2 |
| Tristan da Cunha |  |  |  | 1Dx12;4Ix4 | 4Ax3 | 4Bx1 |
| Campbell Island | 4Ix9;4Jx1 | 2Ax3 | 1Ax3 | 3Ax8;3Ex1;3Fx3;3Gx1 | 3Ax4;4Bx3 | 3Bx1 |
| Auckland Islands: Enderby Is | 4Ix5 | 3Ex3;3Fx1 | 1Ax3 | 3Bx4;3Cx2;3Dx1 | 4Bx6 | 1Ax1;3Ax1 |
| Snares Islands |  |  |  | 4Gx1;4Hx6 | 4Cx5 | 4Bx1 |
| Antipodes Islands | 4Ix10 | 3Dx4 | 1Ax3 | 3Ax8 | 4Bx6 | 3Ax3 |
| Chatham Islands | 4Rx6 | 3Gx1;3Hx4 | 1Bx3 |  |  |  |
